# Imaging natural history museum collections from the bottom up: 3D print technology facilitates imaging of fluid-stored arthropods with flatbed scanners

**DOI:** 10.3897/zookeys.795.28416

**Published:** 2018-11-05

**Authors:** Patina K. Mendez, Sangyeon Lee, Chris E. Venter

**Affiliations:** 1 130 Mulford Hall #3114, Department of Environmental Science, Policy & Management, University of California, Berkeley, Berkeley, CA 94720-3114, USA; 2 Department of Mechanical Engineering, University of California, Berkeley, USA; 3 207 Trinity Road, Brisbane, CA 94005, USA

**Keywords:** digitization, natural history collections, nylon, PLA, Trichoptera

## Abstract

Availability of 3D-printed laboratory equipment holds promise to improve arthropod digitization efforts. A 3D-printed specimen scanning box was designed to image fluid-based arthropod collections using a consumer-grade flatbed scanner. The design was customized to accommodate double-width microscope slides and printed in both Polylactic Acid (PLA) and nylon (Polyamide). The workflow with two or three technicians imaged Trichoptera lots in batches of six scanning boxes. Individual images were cropped from batch imagess using an R script. PLA and nylon both performed similarly with no noticeable breakdown of the plastic; however, dyed nylon leeched color into the ethanol. The total time for handling, imaging, and cropping was ~8 minutes per vial, including returning material to vials and replacing ethanol. Image quality at 2400 dpi was the best and revealed several diagnostic structures valuable for partial identifications with higher utility if structures of the genitalia were captured; however, lower resolution scans may be adequate for natural history collection imaging. Image quality from this technique is similar to other natural history museum imaging techniques; yet, the scanning approach may have wider applications to morphometrics because of lack of distortion. The approach can also be applied to image vouchering for biomonitoring and other ecological studies.

## Introduction

Specimen digitization efforts continue to be a high priority for natural history collections (Baird 2010). Specimens serve as exemplars of morphological variation in taxonomic studies and georeferenced collection records are required to build predictive models of species distributions (Dietrich et al. 2012, Johnson et al. 2013, Shah et al. 2014). When specimens are available digitally, risk of loss and damage to museum specimens can be reduced because only subsets of material may be requested by specialists and available images may facilitate remote identification of unknowns. Although various workflows and custom imaging systems have been developed to make museum material easier to access and manage, imaging museum specimens and digitizing associated information remains acutely laborious and expensive (Vollmar et al. 2010).

**Figure 1.**
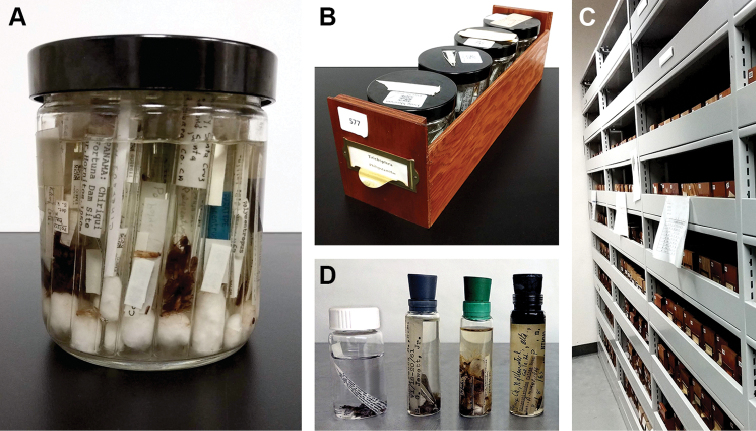
Fluid-stored arthropods in **A** a museum jar with shell vials **B** shell vials in a jar rack **C** racks in storage shelves at the Essig Museum of Entomology. Other fluid-based storage approaches include **D** screw-top scintillation vials and stoppered vials. Individually capped vials are commonly stored in racks on shelves.

Arthropod imaging efforts are largely focused on pinned collections because challenges in imaging specimens stored in fluids is complicated by greater logistical difficulties, making their imaging a lower priority. Soft-bodied invertebrates such as larval insects, spiders, and adult aquatic insects are preferentially stored in ethanol or other preservatives so that shrinking during drying does not distort or obscure morphological structures required for identification. Vials are often stored within larger jars, and each vial often contains multiple specimens in a “lot” sometimes with free-floating dissections (e.g., genitalia) (Figure 1). Multiple labels must be removed from the vial and laid out when imaged. Fluid stored material can be messy and time-intensive to work with, often containing other preservatives that are toxic or noxious, and aged fluids often become yellowed or cloudy over time, requiring their replacement.

These curatorial activities during the imaging process results in longer handling times than pinned material when digitizing, thereby increasing digitization costs (Vollmar et al. 2010). The need to include fluid to support the soft-bodied specimens can make photographic imaging difficult because of surface reflections and distortion from the liquid. However, adoption of a process to image fluid collections is limited by a lack of standardized equipment, defined laboratory workflows, and the perceived inability to image multiple samples at the same time. Scanning material offers an opportunity to digitize fluid material because surface reflections become a non-issue, and scanners lack distortion that occurs in camera lenses because they scan each point by moving across the subject. Given that aquatic species are some of the most threatened under impending climate change (DeWalt et al. 2005, Shah et al. 2014) and their museum records exist primarily in fluid collections, digitization efforts of freshwater species are critical for identifying species and habitats most at risk.

3D printing technology can be used to develop custom equipment for research and can take advantage of available high-quality laboratory materials. Custom laboratory and field equipment is common in the life sciences because the materials are low cost and can be designed for the study goals. Although 3D printing has been common in prototyping in many industries, its adoption in the life sciences has just recently emerged: beetle decoys made on 3D printers can be used to visually attract emerald ash borers (Domingue et al. 2015), life history behavior of insets can be monitored with custom 3D-printed equipment (Berry et al. 2016), and field equipment can be repaired and improved with 3D-printed parts (Will and Steplowski 2016). 3D-printed equipment allows sizes and shapes to be specific to the application and can be designed to interface with existing equipment, be made from a wide range of materials, and compared to custom fabrication, can be relatively low cost.

The goal of this project was to design a standardized 3D-printed piece of laboratory equipment to streamline imaging of small invertebrates stored in fluid collections of natural history museums. Specifically, we designed the system to image on a flatbed scanner using standard, large, optically-clear 75 × 50 × 1 mm glass slides mounted into a 3D-printed plastic box. We also provide a basic scanning procedure with image resolution settings and an R script to subset the batch image scans. We tested our system using fluid-stored material from the Essig Museum of Entomology. We imaged specimen lots of Trichoptera because the wide range of body sizes and life forms provide a robust test of conditions found in wet collections.

## Methods

### Scanning box design and construction

To standardize the box size and reduce the need to cut custom glass, we designed the scanning box around a pair of large, double-wide microscope slides (75 × 50 × 1 mm, Fisher Scientific, Fisherbrand Plain Microscope Slides 12–550C). We selected this glass type and size because of its clarity, durability, relatively low cost (approx. $0.50/slide) and wide availability.

The imaging design approach addresses issues with imaging in liquid, specifically to eliminate reflections on the surface from overhead shots by scanning through a clear-bottomed box. This design is based on an improvised glass-bottomed VWR pipet box lid-scanning approach used by some researchers for imaging voucher materials as required for the BOLD project (Milton et al. 2013). The final version of the scanning box design was also inspired by a design for photographing aquatic insects using microscope slides to create miniature aquaria for aquatic macrophotography (C Riley Nelson, pers. comm.).

**Figure 2.**
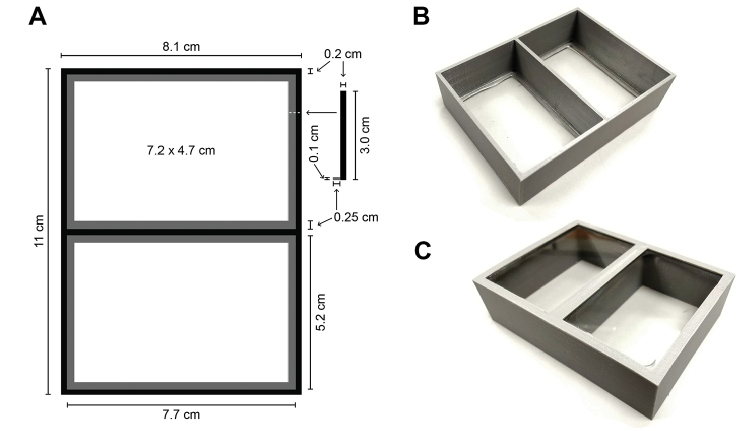
Fluid material scanning box: **A** dimensions of top-down and cross-section view of box plans, and finished box with glass affixed in **B** top and **C** bottom view.

Our approach used 3D print technology because of its ability to produce precise, low-volume runs in a variety of materials. Using Autodesk Meshmixer 2.9 (Autodesk 2015), we designed the box frame to have two panels, one for label information and one for specimens, resulting in a design box size of 8.1 cm × 11.0 cm × 3.0 cm with 0.2 cm walls (Figure 2). A 0.25 × 0.1 cm lip along the interior frame of the box bottom supports the slide once glued. We created the frame design and uploaded the StereoLithography (.stl) file to Shapeways (https://www.shapeways.com) for printing. This STL file is available from (https://github.com/pkmendez/museumscanbox) for download.

To compare frame materials, we printed the design in multiple materials, colors, and finishes at Shapeways. Polylactic Acid (PLA; Natureworks, Ingeo Biopolymer 4043D) designs are printed using Fused Deposition Modeling Technology by extruding melted PLA through a hot tip. Nylon (polyamide, Electro Optical Systems, PA2200) is printed through an ink-jet process and comes in unfinished (print lines visible) and polished (rough sides are abraded by blasting with polishing pellets). We printed PLA models in white, black, and grey (approx. $25 USD each). Nylon was available in white or dyed. We tested black and orange, both unfinished and polished (approx. $28 USD each). Although early prototypes also included prints in ABS-like materials, we did not include this material for further consideration because of the higher costs per print and odors emitted during the materials testing phases. Instead tests were limited to PLA and nylon because of their similar cost and print time.

To complete each box, we fixed the slide into the box with two applications of E6000 silicone adhesive. To apply each layer, we used a wide-tip syringe loaded with adhesive, carefully tracing the margins of the slide and air-dried the box for 24 hours. E6000 shrinks when it dries and we found that two applications prevented most leaks.

### Materials testing

We tested the completed scanning boxes by filling each box approximately 1/3 full of 75% ethanol. We examined the boxes every 20 minutes over the first hour, and then hourly thereafter for 4 hours. We recorded any leaking, softening of the material, staining, fading of colors, or odors.

### Scanning workflow

**Figure 3.**
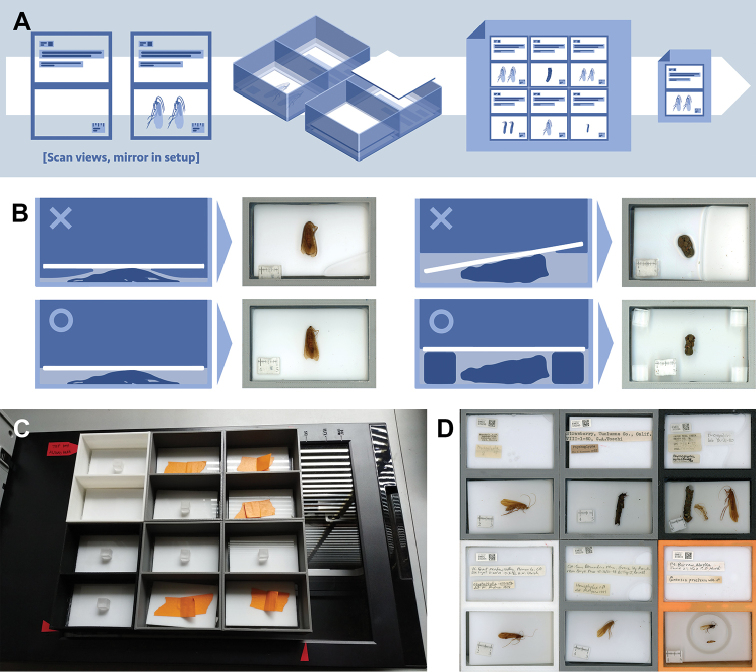
Scanning process images: **A** scanning process with scan view setup, box covers, full scan, and cropped image **B** required fill levels of fluid for box covers to prevent bubbles and standoffs (left) to prevent an uneven scan (right) **C** six boxes set up on scanner; and **D** resulting full scan. Clear Plexiglas cube standoffs **B** and slip-joint rings **D** (lower-right) improve scans.

**Figure 4.**
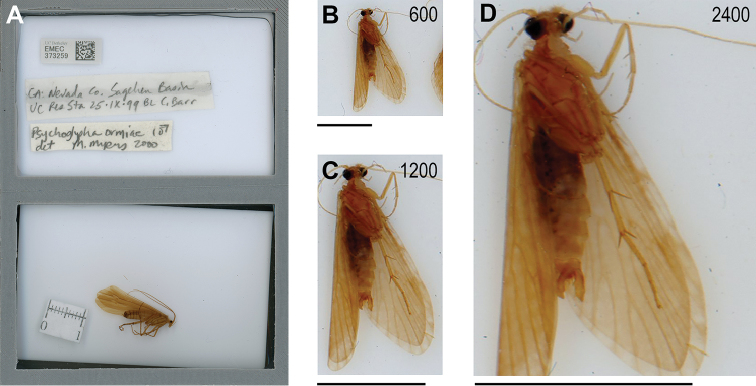
Cropped scan and scan resolution comparison: **A** final cropped scan of EMEC 37259, *Psychoglyphaormiae* (Ross, 1938) ♂, with standard layout of labels and specimens **B–D** comparison of scan resolution settings of 600 dpi, 1200 dpi, and 2400 dpi to show 100% scale display quality differences. Black scale bar is 5 mm for *Rhyacophilaharmstoni* Ross, 1944 ♂ (EMEC 41255, NV: White Pine Co.) in all images. Images are unmodified in software (e.g., no sharpening, white balance, or color correction).

The scanning workflow consisted of moving the specimen to the scanning boxes, placing them on the scanner, arranging and covering the specimens, acquiring a batch image, and then cropping the images in software (Figure 3A). To standardize scanning of the collection in the Essig Museum of Entomology similar to the CalBug project (CalBug 2017), we placed all labels into the upper panel of the scanning box with a unique Essig Museum of Entomology Catalog label (2D DataMatrix code) in the upper left corner of the box, followed by the collection label(s), then any determination, count, and habitat labels (Figure 4A). We covered the labels with a 4.8 × 7.3 cm piece of 1/8” thick acrylic Plexiglas in “sign white” (Tap Plastics, $1 each) to hold down the labels and serve as a background. In the lower panel, we arranged the specimens so that they did not overlap, added a 10 mm scale bar in the lower half of the panel, and covered the specimens with a white Plexiglas background. To both the label and specimen boxes, we added ethanol to submerge the white background and reduce imaging distortions caused by air bubbles (Figure 3B). We also replaced yellowed ethanol when necessary resullting in notable improvement to scans.

To improve the quality of the scans, we arranged specimen strategically and added supports in the specimen box. For larvae, we lined up the caddisfly cases, but did not remove larvae from cases, and for adults with genitalic dissections, we often paired the body and dissections within a ring (a plumbing slip joint washer) to keep the material from floating and to serve as a standoff for the background (Figure 3D). For larger specimens, such as large-cased limnephilids, we used polished Plexiglas cubes 3/8” on a side in each corner of the panel to keep the white background panel from rocking (Figure 3B). To prevent buildup of oils on the scan boxes, we washed the scanning boxes in tap water and dish soap at the end of each daily scanning session and left them to air dry overnight. Before beginning the next scanning session, we cleaned both the scanner glass and the exterior slide glass of the scanning box using household glass cleaner.

To image specimens efficiently, and reduce overall handling time, we imaged batches of 6 boxes (Figure 3 C, D) on an Epson Perfection C600 Photo Scanner connected to a workstation (Lenovo Ideacentre Q190, 1.60 GHz Intel Celeron, 4GB Ram, Windows 8). We arranged the boxes in a compact 2 × 3 grid with no spacing between boxes. The set of boxes was aligned with tape-marks on the scanner for the outer boundaries (Figure 3C) and we set the marquee scanning area to capture only the 6 boxes in one scan. Once set, this marquee box did not need to be repositioned between scans.

To determine the most appropriate resolution and file size to image batches, we scanned a representative batch at 600 dpi, 1200 dpi, and 2400 dpi, compared file characteristics and determined the resolvable structures at each resolution. To determine resolvable structures, we compared images of *Rhyacophilaharmstoni* Ross, 1944 (UC BERKELEY EMEC 41255), a caddisfly with an overall adult body length of approximately 1 cm, but also examined other images scanned at 2400 dpi to identify resolvable structures from other species. Although higher dpi options were available for the scanner, settings above 2400 dpi exceeded the capabilities of the workstation and file sizes became too large to process efficiently. For the final archived version, we scanned each batch at 2400 dpi in color and saved images as JPG files. In early phases of the project, we saved images as TIFF files, but file sizes were too large to store and process. For most scans, we pre-scanned to check label and specimen positioning with a preview.

Imaging technicians worked in teams of 2–3 for 3-hour scanning sessions with a set of 12 scanning boxes in two batches of 6. 1–2 technicians loaded batches of scanning boxes by working one vial at a time to load the label panel and the specimen panel. Fluid-stored material in the Essig Museum of Entomology is stored in thin, long shell vials (mostly 8 × 90 mm and 12 × 90 mm) stoppered with a firm piece of cotton and inserted cotton-side down in wide-mouth pint jars of ethanol. Each sample scanned required that a vial was selected from pint jar, the cotton was removed, and the contents of the vial emptied into the scanning box, and the vial rinsed with ethanol to make sure all small parts were moved to the scanning box. A batch was then passed to a technician who operated the scanning station.

The scanning technician moved the boxes to the scanner, arranged and covered the specimens, previewed the scans and then completed the high-resolution scans. While the scanning technician scanned the batch, the loading technicians either loaded a new batch if it was the first run, or unloaded the batch that came off the scanner, and then loaded the new batch. Unloading the scanning boxes required technicians to carefully return the material and labels (including the new 2D DataMatrix code label) to the shell vials, completely search the scanning boxes for small parts, refill ethanol, re-stopper the vials with cotton and return them to a pint jar. As part of this process, we often curated the material by replacing ethanol, replacing chipped shell vials, and re-copying faded labels. But, we avoided replacing all ethanol in every sample to reduce the chance of losing small parts of specimens. The estimates for the handling time include these activities.

### Image processing

To process the 6-box batch image into a single file for each image, we bulk-processed the 6-panel images using an R script (available from: https://github.com/pkmendez/museumscanbox) (R Core Team 2016) using package ‘magick’ (v. 0.4) (Ooms 2017). We loaded each image, cropped the image into 6 separate files, rotated the image to portrait, and renamed each file as a subname of the original image. The script uses the dimensions of the marquee panel to determine where to crop the image to make 6 equivalent panels. We did not perform edge detection or rotate each image by hand in software, instead relying on high-quality, well-aligned scans with accurate marquee boxes. Because of the size of each of the images, we increased the memory limit of our R workspace to 20000 MB, rotated after cropping, and removed all images from the R workspace after each loop iteration.

We later manually renamed files to the Essig Museum naming convention (e.g., EMEC41255 Rhyacophilaharmstoni.jpg). To keep archive images directly comparable and avoid the need to recalibrate scale bars between photos for morphometric measurements, we did not standardize the archive image dimensions, instead leaving each at the native dimensions after cropping. We also did not make any color corrections to the images to avoid increasing processing time by having to manually interact with each cropped scan. Images were resized to be comparable in dimensions to images of the pinned material and deposited into the Essig Museum of Entomology’s custom MySQL database and later transcribed in the workflow along with images of pinned material.

## Results

### Materials performance

Of the three materials tested, PLA performed best with no reaction to the ethanol during the 4-hour test with leaks only occurring when the slide did not form a good seal with the adhesive (Table 1). We sealed leaks with an additional application of E6000 at the slide perimeter. Polished white nylon performed acceptably, with no softening of materials or odors, yet some ethanol eventually moved up and over the sides because of the surface texture. This effect occurred more quickly in the unpolished nylon because it has a rougher surface, increasing the surface area for the liquid to cling. The nylon boxes in orange and black leeched the dye used to stain the boxes, staining the ethanol. Because we aimed to avoid staining collection material from the mobilized stain, we only recommend nylon in white.

**Table 1.** 3D materials and response to 4-hour ethanol test. PLA (white, black, and grey) and nylon (white, black, orange, both unfinished and polished versions) exposed to 75% ethanol.

Material	Material Information	Performance in EtOH test
**PLA**	Fused Deposition Modeling Technology. Heat-extruded, hard plastic, unpolished. Lines visible between layers.	No odors, softening, leaking, or creep of ethanol. No color change to model or ethanol.
**Nylon (strong and flexible)**	Inkjet-like process, requires support material when printing, unfinished or polished. Unfinished noticeably rougher (layer lines) than polished nylon.	No odors or softening. Creep of ethanol up and over side. Extreme leeching of color into ethanol in black and orange models.

### Scanning size and quality

**Figure 5.**
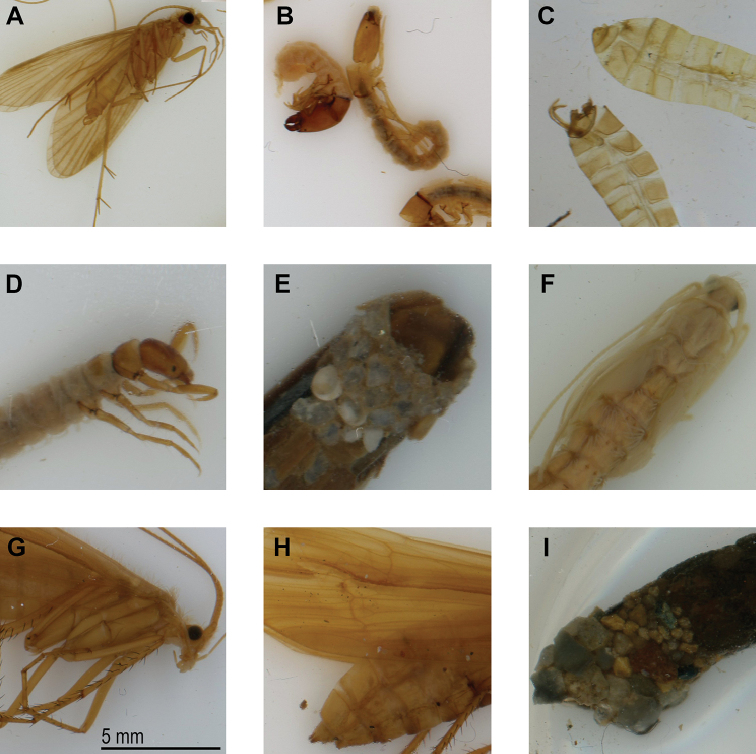
Example scans of different species and life stages of Essig Museum of Entomology Trichoptera showing detail of morphological features **A***Dolophilodesnovusamericana* (Ling, 1938) ♂, CA: Marin Co., EMEC 373433 **B***Wormaldia* sp. larvae, CA: Nevada Co., EMEC 1194742 **C***Limnephilusfrijole* Ross, 1944 genitalic dissections of ♀♂, CA: Modoc Co., EMEC 373223 **D** larva and **E** case of *Yphriacalifornica* (Banks, 1907), CA: El Dorado Co., EMEC 373355 **F**Limnephilidae pupa, no location data, EMEC 373316 **G***Psychoglyphaormiae* ♀ (Ross, 1938), CA: Nevada Co., EMEC 373259 **H***Psychoglypha* sp. ♀, CA: Nevada Co., EMEC 373266 and **I***Hesperophylaxdesignatus* (Walker, 1852) case, CA: Mono Co., EMEC 373246. All scans are unmodified in software (no sharpening, white balance, or color correction). Scale bar: 5mm.

Scanning time and file size increased with increasing dpi setting on the scanner (Table 2). Scan times ranged from <15 s to 3.4 m. File size for TIFF were ~25–37× larger than JPG files at the same dpi. File size at the highest resolution of 2400 dpi for JPGs was 16× larger than the 600 dpi scan. All resolutions recovered basic features such general wing venation, tibial spurs, and determining if individuals were likely males or females based on the shape of the abdomen if visible through the wings (Figure 4). As resolution increased, the detail increased on all structures and allowed for individual segments of maxillary and labial palps, antennae and tarsi to be identified, ocelli presence and location, setae and spines (e.g., head, thorax, wings, legs), sutures and individual segments of thorax and abdomen, and shape of larger structure of male genitalia to be discerned at the highest resolution (Figs 4B–D, 5C). In general, scans at 2400 dpi were the clearest. In most cases, however, family-level diagnostic features such as setal warts on the head and thorax were not apparent, often because of positioning of the specimen. In some cases, images were informative to identify some specimens that were missing determinations in the collection.

**Table 2.** Scanning details and resolvable structures of Trichoptera at different resolutions. All dimensions are approximate for one set of 6 boxes in one scanning pass.

Scanning resolution (dpi)	Scanning time	Dimensions (pixels)	JPG file size	TIFF file size	Resolvable Structures in adults
600	< 15s	5091 × 3714	3.4 MB	83.2 MB	Tibial spurs, wing venation major veins, abdominal shape to determine sex, large male genitalic structures.
1200	1m 10s	10183 × 11428	10.2 MB	333 MB	The same structures @ 600 dpi, but greater detail on shape and count of tibial spurs, detail in wing pigment, shape detail on male genitalic structures, shape of maxillary and labial palp segments sometimes possible to discern.
2400	3m 40s	20366 × 22856	34 MB	1.29 GB	The same structures available @ 1200 dpi, but also segments of antennae, individual setae on the head and abdomen, and additional detail on male genitalic structures. Tibial spines, ocelli, and setae on wings was also discernable for some taxa.

For larvae, materials and structure of the case were identifiable (Figure 5E, I), as well as head shape, mandibles and some leg features (Figure 5B, D). Cases often obscured abdominal detail and we did not remove caddisflies from cases to keep material associated and minimize disruption for later activities for experts. For pupae, abdominal gills and hook-plates, as well as facial setal warts and other features of segments were visible (Figure 5F).

Scan quality decreased when scanner glass and exterior surfaces of slide glass were not cleaned and dried between sessions resulting in condensation on the slide surface. Yellowed ethanol also made for poor scans. When specimens were large (e.g., cased limnephilids), scans were darker than shallower scans. Dark-bodied specimens often lacked detail.

### Scanning workflow

When technicians worked in teams of two or three, they scanned 3–4 batches/hour (12–20 mins per batch) resulting in a rate of ~7 mins/vial/worker from the unloading step through rehousing the material, including routine curatorial activities. Although increasing the team to three members increased the speed of loading and unloading, the scanning step was often the time limiting step in the process because the specimen had to be arranged and previewed before scanning.

### Image processing

Large file sizes increased image processing times often exceeding the available memory on our scanning computer and we had to perform most image processing on a computer with higher specifications (Razer RZ09-01302E21, 2.60 GHz Intel Core i7-4720HQ, 16GB Ram, Windows 10). Processing took ~1.5 mins to cut and rotate the 6 individual scanning box images per batch resulting in individual files of 5MB each (~3.5–7.5 MB). Alignment issues occurred occasionally when the marquee was misaligned in the bulk scan as a result of technician error. These errors were sometimes correctable by realigning and hand-cropping in image editing software.

## Discussion

Using 3D print technology in research workflows is an opportunity to reduce costs for customized laboratory equipment as well as provide standardized and reproducible components available anywhere that it can be printed. For imaging arthropod natural history collections, low-cost PLA and nylon performed well in a bulk scanning workflow. Images were more than adequate for museum documentation and also recorded features at a high enough resolution for a partial identification of Trichoptera in some instances. We recommend this equipment and workflow as a low-cost method to image natural history museum fluid collections.

### Performance of scanning box

Valued for its wide availability on consumer-level 3D printers, PLA is a desirable material for fabrication of the museum scanning boxes because of the low cost (~$2/box on home or campus printers). When printed at 100% infill, leaks between layers never occurred. As a material advertised for its biodegradable and compostable properties, the key concern as a material for the scanning box would be degradation of the PLA over time as a result of its interaction with the 70–75% ethanol solution. Hydrolysis of PLA into lactic acid occurs when the material interacts with ethanol and water with increased breakdown occurring more at lower concentrations of ethanol (Iñiguez-Franco et al. 2016). Lactic acid is commonly used at its 85% reagent strength concentration at 130 °C when clearing male genitalia of caddisflies because it dissolves soft internal abdominal structures and loosens phallic structures critical for species identifications (Blahnik et al. 2007). During this process, the lactic acid is rinsed from the structures completely before the specimen is returned to ethanol for curation.

We hypothesize that lactic acid resulting from the breakdown of PLA during the short scanning sessions, often occurring with ethanol changes when rehousing the specimens, should minimize retention of lactic acid or at least keep at it low levels similar to rinsed specimen after the clearing process. In the scanning box application, room temperatures are much lower than the 40 °C temperature of PLA hydrolysis conducted by Iñiguez-Franco et al. (2016). We doubt that any resultant lactic acid would continue to clear specimens once they have been permanently stored. Moreover, lactic acid generally does not affect recovery of DNA from specimens that have been exposed to it (Paquin and Vink 2009). Although field studies at high summer temperatures report color changes in PLA over months of outdoor exposure (Will and Steplowski 2016), the scanning boxes for this project remained unchanged after 1.5 years of use at room temperatures in the laboratory.

Nylon (polyamide) serves as a viable alternative because of its wide availability and chemical resistance for the non-dyed models. However, we did not have access to printers that printed in nylon other than through web vendors, limiting our ability to inexpensively prototype and test, making it the more expensive of the two materials. Nylon scanning boxes remained stable after 1.5 years of use in the laboratory.

### Imaging efficiency and workflow

The efficiency of imaging through a scanning approach was similar to other fluid-based approaches yet required double the handling time of pinned material. Volunteers working in teams of 2–3 processed vials at a rate of ~8 vials/worker/hour (~7 mins per vial + <1min/image of digital processing) from unloading and positioning specimen to scanning and rehousing. This rate is similar to imaging samples from ichthyology and herpetology museums, where 41% of the museums reported 6–10 mins/sample and 22% reported 11–15 mins/sample (Vollmar et al. 2010). However, fluid sample scanning was slower compared to imaging pinned material. The latter ranging from 1.3 mins/museum object for the CalBug project (P Oboyski, pers. comm.) to 3–4 mins/pinned specimen including short data entry at the Australian Museum (Flemons and Berents 2012). This difference in handling time for fluid compared to pinned material results from the time required to remove the material from the vial, position the labels and specimens on the scanner, and then curate and rehouse the specimens. Over tens of thousands of specimens, this summed small difference in overall time required to image a collection may be on the order of years or decades, depending on the size of the collection.

Compared to whole drawer imaging approaches such as SatScan (Blagoderov et al. 2012), DScan (Schmidt et al. 2012) or GigaPan (Bertone et al. 2012), imaging the fluid-based collection is considerably slower. Yet the resolution of the information associated with each specimen is much finer-grained and can serve broader functions. All labels are spread out for each sample lot compared to the whole drawer which may have stacked labels and obscured information (Dietrich et al. 2012). Identifications often include the year identified in the handwriting of the worker—this handwriting can be diagnostic for estimating the accuracy of the determination (Flemons and Berents 2010). Unfortunately, other approaches such as multi-vial imaging on a scanner used at InvertNet (Dietrich et al. 2012) could not be applied to the Essig Museum collection because label information is often obscured: up to 40 narrow vials closed with cotton are bulk stored in lots of 20–40 in pint jars (Figure 1A).

### Image quality

Scanning at 2400 dpi created high-quality scans appropriate to resolve some taxonomically-informative structures in many groups of Trichoptera. In most images, diagnostic structures such as tibial spurs, and labial and maxillary palps were clear enough to provide basic level information that is helpful for narrowing-down to the family level, with the exception of dorsal features of the head and thorax. Ocelli and dorsal setal warts were visible if the specimen was positioned on its dorsum for scanning. Some wing venation was clear enough to see major veins, patterning, and scale-like setae, but this detail is certainly not enough for wing-keys that rely on detail such as miniscule hooks and hind-wings (e.g., Ruiter 2000). Unfortunately, Trichoptera preserved in ethanol are often faded or discolored and color was unreliable for identification. Scans of darker-bodied specimen often had contrast issues that may need to be corrected in digital editing software if used for purposes beyond recordkeeping. In most cases, the wings obscured detail on the abdomen, but Trichoptera wings are generally transparent and it was often possible to estimate if the specimens were male or female based on the shape of the abdomen. For specimens with cleared genitalia, some diagnostic characters on genitalia were visible, however for the vast majority of taxa, identification can only be determined by viewing structures of cleared genitalia under a compound microscope, and no mass digitization imaging technique to date would be able to accomplish this task.

Scans have the potential to be used beyond identifications and museum record-keeping. Wing measurements derived from imaging pinned specimens with a camera lens are not reliable for morphometric analyses, especially for larger specimens where error increases if the wing is not aligned with the measurement plane or if there is distortion from the lens used to acquire the image (Hall et al. 2014, Trueman and Yeates 2015). However, caddisfly images acquired by scanning may have value for morphometric analysis of forewings because they are imaged flat and their measurements may be comparable to slide-mounted forewings. In this scanning technique, all images are at the same scale and measurements can be made on all images with a single calibration. Unfortunately, wings were sometimes curled and folded in the vials, and the overlap between wing layers may make it difficult to determine the correct wing to measure. Given the high image resolution and data quality, we expect that these research grade images should be able to be identified digitally when integrated into resources such as iNaturalist or other systems that use machine learning algorithms.

### Considerations for scanning conditions based on application

Although 2400 dpi produced the highest quality scans, the final selection of the scanning settings should be determined based on the desired utility of the scan and how the image files will be stored. For natural history museums, if the scans are primarily a snapshot of the material that provides visual verbatim label information and the count and condition of loan material, a lower resolution and smaller file size may be more desirable. However, acquiring lower resolution scans may not measurably speed up the imaging process because the handling time per vial is so high. If wider utility is desired, such as for identification, teaching, or morphometrics, higher resolution scans are worthwhile. We approached this project from the perspective of wanting to determine the highest quality scans we could get though the scanning method. In our case, the size of the Essig Trichoptera Collection is relatively small (~2,500 sample lots) compared to other fluid collections. We scanned at the higher resolution for longer term research purposes, and later batch downscaled images for museum archiving and display images. These downscaled images are comparable in size and resolution with those taken for the pinned collection with much lower file storage requirements.

The scanning box applications are not limited to museum imaging and this method may serve as a tool that can be used more widely to archive aquatic ecology specimens. For example, samples taken for biological monitoring are not often stored long term, representing a lost opportunity to permanently archive life history condition (e.g., body size, life stage) of aquatic taxa which may ultimately be useful in long-term ecological studies. Images may also be able to provide easily examinable records when physical material is unavailable or as part of a workflow for QA/QC by remote reviewers or experts. Other fluid-based bulk samples such as those from malaise or pitfall traps can be imaged and sent to experts to share samples. This method is appropriate for image-based vouchering to accompany DNA sequences such as those archived by BOLD (Milton et al. 2013).

The design size for this fluid-based application for natural history arthropod collections was based on the wide variability in label sizes for fluid-based collections. Compared to pinned collections, which usually have much smaller labels (e.g., 1.5 cm x 0.75 cm) to fit under the insect body to maximize the number of individuals that fit in a museum drawer, fluid collections have no such constraint. The size of vials used for storage in collections range from wide scintillation vials with caps, to stoppered vials, to very thin shell vials. Many labels tend to be larger because of the need to write by hand with pencil and archival pigment inks to prevent loss of label information in ethanol. The double wide museum slide best accommodated the label and specimen lots of collection material, yet a standard slide sized scanning box may be more appropriate for other collections.

The scanning boxes are a 3D printable design, and with software the design can be modified to change the dimensions of the scanning box for alternative imaging purposes. Smaller scannable areas, such as those designed around a standard microscope slide or even microscope cover slips, may be viable for digital archiving, especially for projects such as BOLD vouchering where label information is not required and the frame needs to only accommodate one specimen; the 3D-printed boxes may be single and mobile, or have a fused layout for batch imaging could match indexing of DNA samples to reduce laboratory errors. The number of images captured would be dependent on the printing size capabilities of the 3D printer and the overall scan area. Larger area scans, such as for large benthic samples or ichthyology, are also possible but there may be challenges in finding high-quality glass that is thin enough not to reduce image quality and inexpensive enough to be used in this application.

## Conclusions

The value of developing a standardized method and equipment for imaging fluid-based museum material should not be underestimated. In natural history collections, freshwater aquatic species, with the exception of odonates, are some of the least documented with digital images, mainly restricted to images of pinned adult forms. Most museums have not embarked on digitizing fluid material, and this method represents a clear opportunity for a low-cost approach that can be integrated into existing workflows. The scanning box and laboratory workflow performed well with few leaks and used a simple approach for technicians. Compared to other systems, the components are inexpensive, available, and replaceable. Boxes can be printed in-house or ordered with the cost of 12 boxes (2 batches) for well under $400 USD and can be used with existing scanners and computers. Because the design is fairly simple and designed to make use of available laboratory materials, it can be made to accommodate smaller slides or customized to the imaging needs of the museum or for other research needs.
